# A Hybrid Spectral Clustering and Deep Neural Network Ensemble Algorithm for Intrusion Detection in Sensor Networks

**DOI:** 10.3390/s16101701

**Published:** 2016-10-13

**Authors:** Tao Ma, Fen Wang, Jianjun Cheng, Yang Yu, Xiaoyun Chen

**Affiliations:** 1 School of Information Science and Engineering, Lanzhou University, Lanzhou 730000, China; matlzu@163.com (T.M.); chengjianjun@lzu.edu.cn (J.C.); yuyang_1102@163.com (Y.Y.); 2 School of Mathematical and Computer Science, Ningxia Normal University, Guyuan 756000, China; wangfen_2015@163.com

**Keywords:** intrusion detection system, deep neural network, ensemble model, wireless sensor network, spectral clustering

## Abstract

The development of intrusion detection systems (IDS) that are adapted to allow routers and network defence systems to detect malicious network traffic disguised as network protocols or normal access is a critical challenge. This paper proposes a novel approach called SCDNN, which combines spectral clustering (SC) and deep neural network (DNN) algorithms. First, the dataset is divided into *k* subsets based on sample similarity using cluster centres, as in SC. Next, the distance between data points in a testing set and the training set is measured based on similarity features and is fed into the deep neural network algorithm for intrusion detection. Six KDD-Cup99 and NSL-KDD datasets and a sensor network dataset were employed to test the performance of the model. These experimental results indicate that the SCDNN classifier not only performs better than backpropagation neural network (BPNN), support vector machine (SVM), random forest (RF) and Bayes tree models in detection accuracy and the types of abnormal attacks found. It also provides an effective tool of study and analysis of intrusion detection in large networks.

## 1. Introduction

Networking has become ubiquitous in people’s lives and work; hence, network security bears increasing importance for network users and operators. Network intrusion detection is a network security mechanism designed to detect, prevent and repel unauthorized access to a communication or computer network. IDSs play a crucial role in maintaining safe and secure networks. In recent years, vast amounts of network data have been generated due to the application of new network technologies and equipment, which has led to declining detection rates. The intrusion detection process is complicated due to the dynamic nature of networks and the power available for processing high volumes of data from distrusted network systems, making it difficult to achieve high detection accuracy at rapid speeds [1].

Many researchers have proposed innovative approaches to intrusion detection in recent years. These methods, based on detecting behaviour and access resource type, are divided into four categories. The first uses statistical analysis to detect attack types based the relationships between nodes in a network, such as with Bayesian [2], decision tree and fuzzy logic models. These techniques build models of normal network data and calculate the probability that a given sample deviates from these models. If the probability that a sample value’s deviation is greater than threshold, then the sample is classified as a type of attack [3]. The second category is an anomaly detection approach in which most methods require a set of standard normal datasets to train a classifier and determine whether new samples fit the model. Examples of such methods include SC, self-organizing map (SOM) and unsupervised support vector machine approaches [4]. The third category employs classification techniques to detect attack types by taking advantage of machine learning algorithms such as SVM [5], RFs [6], genetic algorithms (GA) and artificial neural networks (ANN), which can prioritize solutions to certain problems. The last category includes hybrid and ensemble detection methods that integrate the advantages of various methods in order to increase detection accuracy [7]. These approaches include bagging, AdaBoost [8], and the Particle Swarm Optimization (PSO)-*K*-means ensemble approach [9]. The PSO-*k*-means method can achieve an optimal number of clusters, as well as increasing detection rates while decreasing false positive rate, and can be successful in detecting network intrusion. In addition, the SVM-KNN-PSO ensemble method proposed by [10] can obtain the best results. However this work is based on binary classification methods, which can distinguish between only two states.

Recently, deep learning has become a popular topic of research, and methods based on deep learning have successfully been widely applied in image identification and speech recognition. In recent literature, deep learning has been used in network security detection. The self-taught learning (STL) model, based on deep learning techniques, was proposed for network intrusion detection. This model utilized a sparse auto-encoder (SAE) to learn dataset features; the NSL-KDD dataset was used to test its detection performance [11]. Deep belief networks (DBN) are used to initialise the parameters of DNN through a pre-training process. Initialising a DNN with probability-based feature vectors is used to detect vehicular networks and significantly improves detection rates [12]. Fiore et al. [13] proposed a novel model for network anomaly detection based on a semi-supervised discriminative restricted Boltzmann machine (DRBM), which decouples training and generalizes the neural networks. In a previous hybrid model, Salama et al. [14] proposed a combination of a DBN and SVM. The DBN is used to reduce the dimensionality of the input dataset and the SVM is used to classify attack types.

For complicated network attacks, some research has focused on rule-based expert detection, which utilizes pre-designed processing rules to detect attacks [15]; however, for very large datasets, rule-based expert approaches become less capable. Additionally, wireless sensor networks (WSN), a new post-Internet network architecture, are widely and increasingly used in a variety of smart environment applications. Adding these to conventional and mobile networks, and taking into consideration the diverse intrusion methods available to malicious agents, the technical requirements of intrusion detection are more complex than ever before [16]. Attacks on WSNs are easier to carry out than those on wired networks because the distribution of WSNs is inherently limited, and because of the multi-hop communication, bandwidth and battery power used therein. Therefore, designing an effective IDS for WSNs is very important. Due to this new environment, new attacks have been devised (e.g., black hole, sink hole, abnormal transmission and packet dropping attacks). The attacks are divided by mechanism into signature, anomaly and hybrid intrusion detection methods. The anomaly detection method, for instance, is widely used for security in WSNs [17].

However, intrusion detection has been insufficient in several ways. First, the learning capacity of traditional detection approaches that sum features of the raw data, map them into vectors, and then feed them to a classifier is limited. When network structure is complicated, learning efficiency further decreases. Second, this primary method only partially represents one or two levels of information; it is insufficient for identifying additional attack types. Third, in real network datasets, the types of network intrusion are similar to those in normal datasets, which confine classifiers from having enough information with which to categorize them. Next, the intrusion actions behave unpredictably, which causes IDS to make costly errors in detecting intrusions. Therefore, it is necessary to find an effective intrusion detection method [18]. Finally, the variety of network types has generated large-scale data with high-dimensional structures, for which traditional intrusion detection approaches are unsuitable [19].

Literature concerning fields in which many-layered learners represent network dataset features is quite limited. Lin et al. [20] proposed combining the *k*-means and *k*-Nearest Neighbour (KNN) algorithms to select dataset features and to categories network attack types. Samples are reconstructed into clusters and a two-dimensional vector dataset is generated with different features. Then, the training dataset is divided into subsets by the KNN algorithm. This approach more efficiently detects normal data and Denial of service (DoS) attacks with high accuracy, but it does not perform well in detecting user-to-root (U2R) and remote-to-local (R2L) attacks, which means that the selected features represent these attack types poorly and the proposed model can only learn limited patterns.

Taking the above into consideration, this paper proposes the SCDNN model, which uses spectral clustering to capture raw data features and divide the dataset into subsets. Each subset is then fed to a DNN. These DNNs can learn information about different characteristics of the subset. The test datasets have previously been divided by training dataset features into subsets. Finally, the test subsets are fed to the trained DNN for intrusion detection. Because the SC algorithm can extract similar features and acquire more information from a dataset, the DNN can obtain sufficient information via prior learning, which enables the DNN to familiarize itself with as many of the rules and features of network attack types as possible. Deep learning is believed to be able to extract features from massive and complex datasets; hence, it can solve non-linear problems with complex and large-scale data (e.g., it has been applied in weather forecasting) [21,22]. Experimental results based on the KDD-CUP99 dataset and NLS-KDD datasets show that an SCDNN generates better accuracy and more robust results than other well-known algorithms, and excels in parallel computing [23,24,25,26].

The contribution of this study is as follows: First, we adopt SC to capture the features of complex network datasets, for attack types similar to normal access, using clustering to find features that divide the dataset into subsets with different cluster centres. Second, taking advantage of multi-layer DNNs, attack types can be learned at high, abstract levels of network data and complex network intrusion rules can be found. The intrusion process is becoming more variable and easily disguised as normal network activity. This situation is particularly common in large datasets and complicates the relationship between distributed processing protocols. Knowledge of categories in a dataset is sufficient for the SCDNN model to be able to learn. Finally, our experimental results indicate that the accuracy, detection and false alarm rates of SCDNN are better than those of conventional methods.

The rest of the paper is organized as follows. The related literature concerning IDS is reviewed in Section 2. Section 3 presents a detailed definition of the proposed approach and describes how the algorithm works. Section 4 describes the experimental dataset and illustrates the data preparation, evaluation criteria, results and discussions of experiments. Finally, the conclusions and suggestions for future work are provided in Section 5.

## 2. Related Works

In this section, the spectral clustering and DNN algorithms are briefly introduced. To overcome the limitations of traditional auto-encoders, this study adapts a SAE and denoising auto-encoders (DAEs) for the DNN. IDSs are a classification problem in which dataset features are very important, because the learner acquires knowledge and patterns based on the characteristics of the data. Additionally, the level of feature representation determines a learner’s performance.

### 2.1. Spectral Clustering Algorithm

Spectral clustering uses the top eigenvectors of a matrix derived from the input data and transforms the clustering problem into a graph cut problem. The graph cutting approach clusters data points by attribute such that densely packed points are in same cluster, whereas the sparse are in different clusters. The minimum cut formula is written as:

(1) $$cut\left(x,y\right)=\sum_{i\in A,j\in B}W_{ij}$$

where $w_{ij}$ is the degree of a vertex from *i* to *j*. There are two balanced cut approaches, Ratio Cut and Ncut; spectral clustering is a relaxation of the two. SC is an effective partitioning strategy that includes the normalized cut [27] and Ng-Jordan-Weiss (NJW) algorithms [28]. These algorithms are relaxations of the spectral partitioning problem; the SC algorithm with a Laplacian matrix can be described in Algorithm 1:
**Algorithm 1:** Spectral Clustering Algorithm
**Input**: Dataset, number of clusters *k*, parameter *σ* and number of iterations iter **Output**: the set of *k* clusters /* Note the symbols of “/*” and “*/” represent comments in this algorithm. */1Calculate the affinity matrix $A\in R^{n\times n}$ and define $A_{ij}=exp\left(-∥s_{i}-s_{j}{∥}^{2}/2\sigma^{2}\right)$ /* In which $S_{i}$ and $S_{j}$ are the original data points *i* and *j*, repctively. */2If i≠j then the $A_{ii}=0$3The D is the diagonal degree matrix and computed with elements: $d_{i}=\sum_{j=1}^{n}A_{ij}$ Given a graph *G* with *n* input vertices, the Laplacian matrix L(n×n)=D-A4Find the *k* largest eigenvectors of the matrix *L* and $\left[x_{1}x_{2}\cdots x_{k}\right]\in R^{\left(n\times k\right)}$5Generate matrix *y* by renormalizing each *x* row, $Y_{ij}=X_{ij}/{\left(\sum_{j}X_{ij}^{2}\right)}^{1/2}$6Minimize the distortion of each row *Y* to regard as the point in clustering term $R^{k}$ using any clustering algorithm, such as a distance-based clustering approach.7Finally, the original point $s_{i}$ is assigned to cluster *j* when the row of $y_{i}$ belongs to the cluster *j*.8**return**
*the set of k clusters and cluster centre.*

### 2.2. DNN Algorithm

The essence of the deep neural network is to learn useful features and construct multi-layer networks from vast amounts of training data. Forecast accuracy is improved using DNNs, allowing more information regarding the raw dataset to be obtained. DNN has deep architectures—including multiple hidden layers—and each hidden layer alone conducts a non-linear transformation from the previous layer to the next [29,30]. Through deep learning, proposed by Hinton et al. [31], a DNN is trained according to two main pre-trained, fine-tuned procedures [32,33].

#### 2.2.1. Auto-Encoders

**Figure 1:**
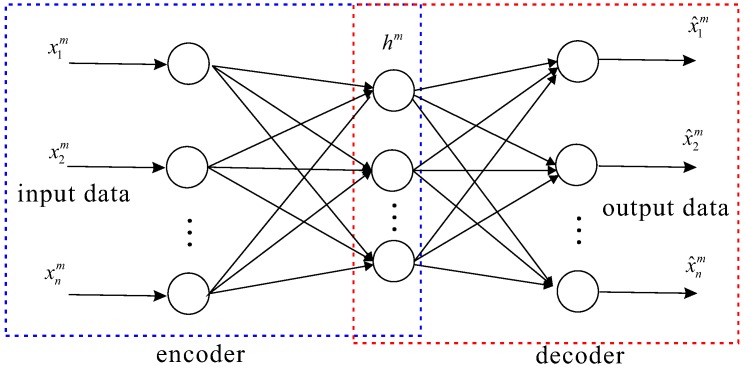
Architecture of an auto-encoder and decoder in a deep neural network (DNN).

An auto-encoder is a type of unsupervised three-layer neural network whose output target is input data [34], which is described in Figure 1. The auto-encoder includes both encoder and decoder networks. The encoder network transforms input data from a high-dimensional space to code in a low-dimensional space, and the decoder network remodels this input from the previous step. The encoder network is defined as an encoding function denoted by $f_{encoder}$. This function details the encoding process:

(2) $$h^{m}=f_{encoder}\left(x^{m}\right)$$

where $x^{m}$ is a data point and $h^{m}$ is the encoding vector obtained from $x^{m}$.

#### 2.2.2. Decoder

The decoder network is defined as a reconstruction function denoted as $f_{decoder}$ and is described as follows:

(3) $${\hat{x}}^{m}=f_{decoder}\left(h^{m}\right)$$

where $x^{m}$ is the decoding vector obtained from $h^{m}$. There are specific algorithms for several encoding and reconstruction functions, including:

(4) $$Logsig:f_{encoder}\left(x^{m}\right)=\frac{1}{1+e^{-x^{m}}}$$

(5) $$Satline:f_{encoder}\left(x^{m}\right)=\left\{\begin{array}{cc} 0 & if\;x^{m}\leq 0\\ z & if\;0<x^{m}<1\\ 1 & if\;x^{m}\geq 1 \end{array}\right.$$

(6) $$Pureline:f_{encoder}\left(x^{m}\right)=x^{m}$$

#### 2.2.3. Sparse Auto-Encoder (SAE)

The default auto-encoder attempts to learn feature approximation, and the function of the auto-encoder of $x^{\prime }$ is close to the raw input data of *x*. It is seems that the identity function is able to learn most features of the input data. However, the number of units in each layer of the neural network is constrained, with $x^{\prime }$ as the minimum original size. When the number of hidden units increases, runtime efficiency decreases for the analysis of input data features, although the auto-encoder will still learn the features of the raw data. From the above analysis, a sparsity coder can constrain the input data with useful identification information [35]. Let $a_{j}^{\left(2\right)}\left(x\right)$ represent the activation of the *j*th hidden units by the auto-encoder from the input *x*, so that the average activation of the *j*th unit in the hidden layer is defined as follows [36]:

(7) $${\hat{\rho }}_{j}=\frac{1}{m}\sum_{i=1}^{m}\left[a_{j}^{\left(2\right)}\left(x^{\left(i\right)}\right)\right]$$

Averaged over the input set, let ${\hat{\rho }}_{j}$ represent the degree of sparsity, where *ρ* is a sparsity parameter. Let ${\hat{\rho }}_{j}=\rho$, which is a small value close to 0; typically, the value of *ρ* is set to 0.05. To satisfy the condition that the activation is near 0, a penalty term is added to optimise the deviation of ${\hat{\rho }}_{j}$ from *ρ*. The optimisation formula is defined as follows:

(8) $$\sum_{j=1}^{s_{2}}\rho log\frac{\rho }{{\hat{\rho }}_{j}}+\left(1-\rho \right)log\frac{1-\rho }{1-{\hat{\rho }}_{j}}$$

in which the number of hidden-layer units is $s_{2}$ and *j* denotes hidden units in the network. The Kullback-Leibler (KL) divergence is written as follows [37]:

(9) $$\sum_{j=1}^{s_{2}}KL\left(\rho ∥\hat{\rho_{j}}\right)$$

where the KL divergence between a Bernoulli random variable with a mean of *ρ* and ${\hat{\rho }}_{j}$ is defined as below:

(10) $$KL\left(\rho ∥\hat{\rho_{j}}\right)=\rho log\frac{\rho }{{\hat{\rho }}_{j}}+\left(1-\rho \right)log\frac{1-\rho }{1-{\hat{\rho }}_{j}}$$

If ${\hat{\rho }}_{j}$ equals *ρ*, then the value of Equation (9) is equal to 0, which means that the KL divergence reaches the minimum value and close to 0, so the overall cost function is defined as follows:

(11) $$J_{sparse}\left(W,b\right)=J\left(W,b\right)+\beta \sum_{j=1}^{s_{2}}KL\left(\rho ∥{\hat{\rho }}_{j}\right)$$

where J(w,b) is the squared error cost function and defined as:

(12) $$J_{sparse}\left(W,b\right)=\left[\frac{1}{m}\sum_{i=1}^{m}J\left(w,b;x^{\left(i\right)},y^{\left(i\right)}\right)\right]+\frac{\lambda }{2}\sum_{l=1}^{n_{l}-1}\sum_{i=1}^{s_{l}}\sum_{j=1}^{s_{l}+1}{\left(W_{ji}^{\left(l\right)}\right)}^{2}$$

The first term of the function J(W,b) is an average sum of squared error over *m* samples and outputs. The second term is a weight decay term which can decrease the magnitude of each weight in a neural network and can effectively prevent overfitting. From Equation (11), *β* is a coefficient to adjust the weight of the sparsity penalty term. The term ${\hat{\rho }}_{j}$ depends on the neural network parameters *W* and *b*, and it is optimised using a derivative checking method [38]. The stochastic gradient descent (SGD) approach is adopted to optimise *W* and *b*, and can be written as follows:

(13) $$W_{ij}\left(l\right)=W_{ij}\left(l\right)-\varepsilon \frac{\partial }{\partial W_{ij}\left(l\right)}C_{sparse}\left(W,b\right)$$

(14) $$b_{i}\left(l\right)=bi\left(l\right)-\varepsilon \frac{\partial }{\partial b_{i}\left(l\right)}C_{sparse}\left(W,b\right)$$

in Equations (13) and (14), *ε* is the learning rate; squared errors are obtained by training over examples and calculating the average ${\hat{\rho }}_{j}$. The best values of *W* and *b* are obtained by back propagation with SGD.

#### 2.2.4. Denoising Auto-Encoders (DAEs)

There is a way to capture something useful about the hidden units with input data. DAEs are used to discover more robust hidden-layer features and can effectively prevent the networks from simply learning features. The DAE is a stochastic auto-encoder that tries to represent information about the input data that can only be learned by capturing the statistical attributes between the encoded and input data. The DAEs can be represented in a variety of ways, including from stochastic operator, bottom-up-information and top-down-generative model perspectives. In this paper, the DAEs learned feature representations by adjusting partial corruption from the input pattern [39]. A fixed number of components in each input x(i) are chosen randomly and their value set to zero; this distribution is called qD(·), then the joint distribution of input and output is defined as follows:

(15) $$q^{0}\left(X,\chi ,y\right)=q_{0}\left(X\right)qD\left(\chi |X\right)\delta_{f_{\theta }\left(\chi \right)\left(y\right)}$$

in which the encoding of *X* is a stochastic mapping by the distribution χ∼qD(χ|X). If the condition $f_{\theta }\left(x\right)\neq y\cdot q^{0}\left(X\right)$ is satisfied, then $\delta_{f_{\theta }\left(\chi \right)}\left(y\right)$ indicates the empirical distribution associated with the input data and set to 0. *χ* can be determined by *y*. In order to obtain the best reconstructed version of $X^{\prime }$ with consideration between *χ* and *y*, let *θ* be a parameter of the joint distribution of Equation (15), and calculate the minimum error of the cost function of the input matrix *X* from *y* as follows:

(16) $$arg\min_{\theta ,\theta^{\prime }}E_{q^{0}\left(X,\chi \right)}\left[L\left(X,X^{\prime }\right)\right]$$

where $\left[L\left(X,X^{\prime }\right)\right]$ is the error function depending on input *x* and the reconstructed $X^{\prime }$. The best cost function value is then obtained using SGD. To encode optimised denoising codes, there is a trick in encoder process. Let *y* be a hidden representation obtained with the function sigmoid(Wχ+b), where *χ* is a generated corrupted version of the input data and the parameters of the function are denoted as θ={W,b}. Each corrupted code contains some elements of input *X* randomly set to 0. Then, the final corrupted version can be minimised in Equation (16).

#### 2.2.5. Pre-Training

*N* auto-encoders can be stacked to pre-train an *N*-hidden-layer DNN. When given an input dataset, the input layer and the first hidden layer of the DNN are treated as the encoder network of the first auto-encoder. Next, the first auto-encoder is trained by minimising its reconstruction error. The trained parameter set of the encoder network is used to initialise the first hidden layer of the DNN. Then, the first and second hidden layers of the DNN are regarded as the encoder network of the second auto-encoder. Accordingly, the second hidden layer of the DNN is initialised by the second trained auto-encoder. This process proceeds until the *N*th auto-encoder is trained to initialise the final hidden layer of the DNN. Thus, all hidden layers of the DNN are stacked in an auto-encoder in each training *N* times, and are regarded as pre-trained. This pre-training process is proven to be significantly better than random initialisation of the DNN and conducive to achieving generalisation in classification [40,41].

#### 2.2.6. Fine-Tuning

Fine-tuning is a supervised process that improves the performance of a DNN. The network is retrained, training data are labelled, and errors calculated by the difference between real and predicted values are back-propagated using SGD for all multi-layer networks. SGD randomly selects dataset samples and iteratively updates the gradient direction with weight parameters. The best gradient direction is obtained with a minimum loss function. The advantage of SGD is that it converges faster than the traditional gradient descent methods do, and does not consider the entire dataset, making it suitable for complex neural networks. The SGD equation is defined below:

(17) $$E=\frac{1}{2}\sum_{j=1}M{\left(y_{i}-t_{i}\right)}^{2}$$

where *E* is the loss function, *y* is the real label and *t* is the network output. The gradient of weight parameter *w* is obtained by the derivative of the error equation.

(18) $$\frac{\partial E}{\partial w_{ij}}=\frac{\partial E}{\partial y_{j}}\cdot \frac{\partial y_{j}}{\partial u_{j}}\cdot \frac{\partial u_{j}}{\partial w_{ij}}$$

With the gradient of $w_{ij}$, the updated SGD equation is defined as:

(19) $$w_{ij}^{new}=w_{ij}^{old}-\eta \cdot \left(y_{j}-t_{j}\right)\cdot y_{j}\left(1-y_{j}\right)\cdot h_{i}$$

where *η* is the step size and is greater than 0, and *h* is the number of hidden layers in the DNN [42]. This process is tuned and optimised by the weight and threshold based on the correctly labelled data in the DNNs. In this way, the DNN can learn important knowledge for its final output and direct the parameters of the entire network to perform correct classifications [43].

## 3. The Proposed Approach of SCDNN

Several classification engines (BPNN, SVM, RF, Bayes and DNN) perform well given the advantages of their algorithms, which can efficiently handle complex classification problems; hence, these models can be successfully applied to intrusion detection. They usually perform poorly when facing the complex randomness and camouflage of network intrusion dataflow. Therefore, in this section, the proposed approach is employed to solve the above problems. First, the training data subsets divide the training process and calculate centre points by SC from each training point. Second, each training data subset is trained by the corresponding DNNs, where the scale of DNNs is the same as the number of clusters. In this way, the DNNs have learned different characteristics from each subset. Third, the testing data subsets are divided into test datasets by SC, which uses the previous cluster centres in its first step, and these subsets are applied to detect intrusion attack types by pre-trained DNNs. Finally, the output of every DNN is aggregated for the final result of the intrusion detection classifiers.

### 3.1. SCDNN

**Figure 2:**
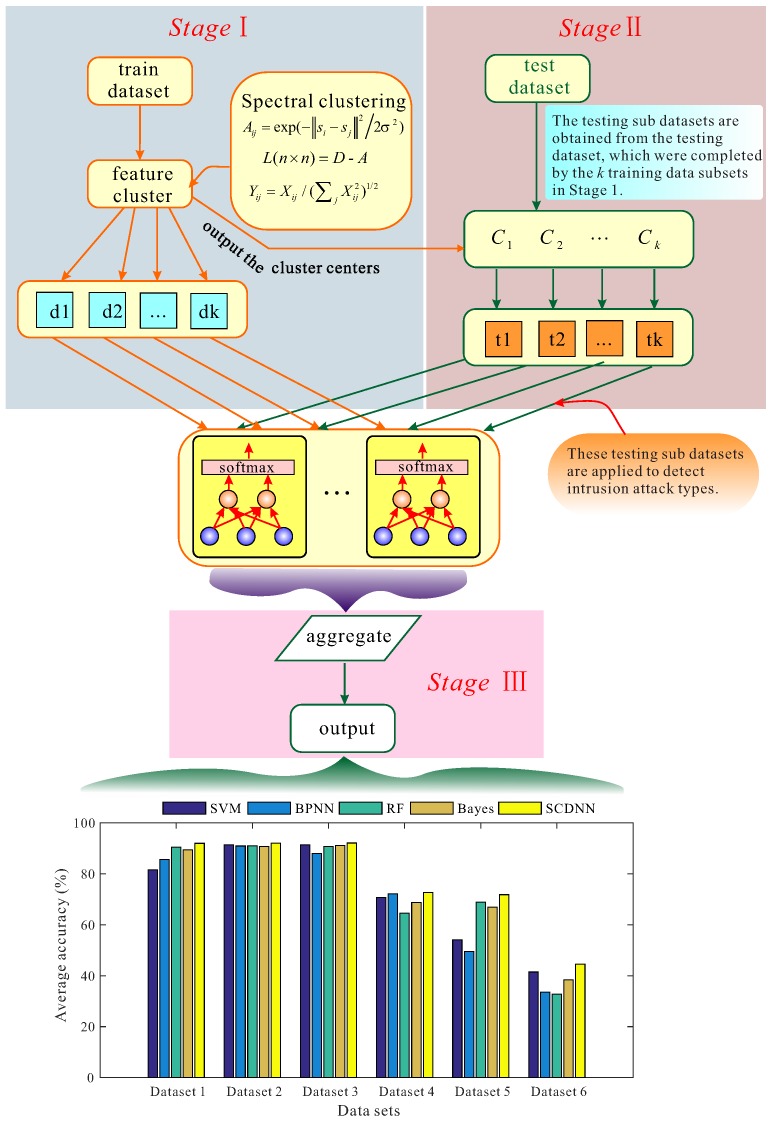
The SCDNN flow chart is divided into three steps and shows each process in detail.

The SCDNN model includes three steps, and the general task in each step is described as follows and shown in Figure 2.

Step 1: The dataset is divided into two components: training and testing datasets. The training dataset is clustered by SC and this output is regarded as the training subset labelled Training Datasets 1–*k*. The SC centres from the training dataset clustering process are stored to serve as initialisation cluster centre for generating the testing dataset clusters. Because intrusion data features indicate similar attributes of each type in the raw dataset, points in the training dataset with similar features are aligned into groups and regarded as the same subset. In order to improve SCDNN performance, different cluster numbers and values of sigma are considered. The number of clusters ranged from 2 to 6 and sigma from 0.1 to 1.0. The samples are assigned to one cluster by similarity. The minimum distance from a data point to each cluster centre is measured by Algorithm 1 and each point is assigned to a cluster. The training subsets generated by clusters are given as input to the DNNs. In order to train the different subsets, the number of DNNs is equal to the number of data subsets. The architecture of each DNN consists of five layers: two hidden layers, one input layer, one softmax layer and one output layer. In this paper, the two hidden layers learn features from each training subset and the top layer is a five-dimensional output vector. Each training subset which are generated from the *k*th cluster centre by SC algorithm in clustering process, regarded as input data to feed into the *k*th DNN, respectively. These trained sub-DNN models are marked as sub-DNN 1 through sub-DNN *k*.

Step 2: The testing dataset, which has been divided from the raw dataset, is used to generate *k* datasets. The previous cluster centres obtained from SC cluster in Step 1, are regarded as the initialization cluster centres of the SC algorithm in this step. The test dataset which are divided by SC cluster, are regarded testing subsets. These subsets are denoted as Test 1 through Test *k*.

Step 3: The *k* testing data subsets are fed into *k* sub-DNNs, which were completed by the *k* training data subsets in Step 1. The output of each sub-DNN is integrated as the final output and employed to analyse positive detection rates. Then, a confusion matrix is used to analyse the detection performance of the five classifications: Normal, DoS, Probe, U2R and R2L.

### 3.2. The SCDNN Algorithm

The SCDNN algorithm adopts a deep learning approach to categorise network attack types and fine-tune its weights and thresholds using backpropagation. The input vectors map low-dimensional space with DAEs and SAE to discover patterns in the data. The SCDNN algorithm is detailed in Algorithm 2.
**Algorithm 2:** SCDNN
**Input**: dataset, cluster number, number of hidden-layer nodes HLN, number of hidden layers HL. **Output**: Final prediction results /*Note the symbols of “/*” and “*/” represent comments in this algorithm.*/1Divide the raw dataset into two components: a training dataset and a testing dataset. /*get the largest matrix eigenvectors and training data subsets*/2Obtain the cluster centres and SC results using Algorithm 1. Here, the clustering results are regarded as training data subsets. /*Train each DNN with each training data subset*/3The learning rate, denoising and sparsity parameters are set and the weight and bias are randomly initialised.4The HL are set two hidden layers, HLN is set 40 nodes of the first hidden layer and 20 nodes of second hidden layer.5Compute the sparsity cost function $J_{sparse}\left(W,b\right)=J\left(W,b\right)+\beta \sum_{j=1}^{s_{2}}KL\left(\rho ∥{\hat{\rho }}_{j}\right)$.6Parameter weights and bias are updated as $W_{ij}\left(l\right)=W_{ij}\left(l\right)-\varepsilon \frac{\partial }{\partial W_{ij}\left(l\right)}C_{sparse}\left(W,b\right)$ and $b_{i}\left(l\right)=bi\left(l\right)-\varepsilon \frac{\partial }{\partial b_{i}\left(l\right)}C_{sparse}\left(W,b\right)$.7Train *k* sub-DNNs corresponding to the training data subsets.8Fine-tune the sub-DNNs by using backpropagation to train them.9The final structure of the trained sub-DNNs is obtained and they are labelled with each training data subset.10Divide the testing dataset into subsets with SC. Cluster centre parameters from the training data clusters are used.11The testing data subsets are used to test corresponding sub-DNNs, based on each corresponding cluster centre between the testing and training data subsets. /*aggregate each prediction result*/12Results are generated by each sub-DNN, are integrated and the final outputs are obtained.13**return**
*classification result = final output*

The eigenvectors of the point matrix and training dataset are generated by the SC output in Lines 1–2, the *k* DNNs are trained in Lines 3–9, the testing data subsets are obtained by calculating distance with the NJW algorithm in Line 10, the testing data subsets are used to test the DNNs, and the final results are predicted by the aggregation in Lines 11–13.

## 4. Experimental Results

These experiments examine and compare SCDNNs with other detection models: SVMs, BPNNs, RFs and Bayes. Six datasets from the KDD-CUP99 and NSL-KDD are used to evaluate the performance of all models. Then, the parameters, number of cluster and DNN weights are discussed and analysed.

### 4.1. Evaluation Methods

In this study, accuracy, recall, error rate (ER) and specificity are used to evaluate the performance of the detection models. The formulas of the above criteria are calculated as follows [44]:

(20) $$Accuracy=\frac{TP+TN}{TP+TN+FP+FN}$$

(21) $$Recall=\frac{TP}{TP+FN}$$

(22) $$ErrorRate=\frac{FP+FN}{FP+TP+TN+FN}$$

(23) $$SPEC=\frac{TN}{TN+FP}$$

A true positive (TP) is a case that correctly distinguishes network attack type, a true negative (TN) shows normal network data classified correctly as normal, a false negative (FN) denotes a case in which an attack was classified as normal dataflow, and a false positive (FP) means that the a normal case was classified as an attack. The accuracy rate shows the overall correct detection accuracy of the dataset, ER refers to the robustness of the classifier, recall indicates the degree of correctly detected attack types of all cases classified as attacks, and specificity shows the percentage of correctly classified normal data. In the above, higher accuracy and recall and lower ER indicate good performance.

### 4.2. The Dataset

To evaluate the performance of our proposed approach, a series of experiments on the KDD-CUP-99 [31] and NLS-KDD datasets [22] were conducted. The 10% knowledge KDD-CUP-99 datasets are used in these experiments, as it is most comprehensive dataset that is widely used as a benchmark for the performance intrusion detection network models. Each record contains 41-dimensional feature data that of various continuous, discrete and symbolic type. This is unsuitable for network data records because it includes four attack categories (DoS, probe, R2L and U2R) [45].

In order to solve the above problem, pre-trained data are processed in three steps. First, the values of three symbolic features are mapped as a series of numeric values ranging from 1 to *N*, where *N* is the total number of symbols for each feature. Second, the KDD’99 dataset includes five classes, normal, DoS, probe, U2R, and R2L, which were mapped onto the numeric values 1–5, respectively [46]. Finally, because of the range of values of field columns of src_bytes and dst_bytes are quite different, in order to find more patterns and more easily handle the proposed method, the range of values of these two fields are mapped onto 0–5000. After data pre-processing, the total number of dataset features was transformed to 65.

The NLS-KDD dataset includes the KDDTrain+, KDDTest+ and KDDTest-21 subsets, which are used for this experiment because the KDD-CUP-99 datasets contain more frequent and harmful attack records. The NLS-KDD dataset has removed surplus records and is more suitable to evaluating the real-world performance of an intrusion detection algorithm. In order to fairly compare the capability of each algorithm, six datasets were randomly generated from the two KDD-CUP-99 and NSL-KDD datasets, which reduces the amount of data [47]. These are labelled Datasets 1 through 6. Probe, U2R and R2L attack classes occur with low frequency in the raw dataset, so these three types were manually distributed, whereas normal and DoS records were randomly selected based on three scales with 1%, 5% and 10% of total in the KDD-CUP-99 dataset, labelled Dataset 1–3, respectively [48].

For the NLS-KDD dataset, the three data subsets are generated from two training and two testing sets and designated Datasets 4–6. The training sets randomly selected 20% and 50% of the raw dataset, and the testing sets are KDDTest+ and KDDTest-21, respectively. Table 1 describes the six new datasets in detail. The training data of the six datasets include 24 attack types, and the testing data contain 38 attack types. Therefore, the test dataset includes specific attack types which are not presented in the training dataset, which is closer to the actual condition of network intrusion. In this way, we can evaluate the ability of each model to find new attack types.

The six new datasets are used to evaluate the performance of the SCDNN and to compare it to the other detection methods previously mentioned. In addition, these experiments evaluate model performance for one of the novel network architectures arising in today’s networking environment, such as ad hoc wireless networks, WSNs and wireless mesh networks. These networks have developed rapidly and are easier to attack than wired networks. In order to determine the detection performance of the proposed model in WSNs, the following experiments were carried out [49].

### 4.3. SCDNN Experiment I

In this section, the number of clusters *k* in SCDNN is evaluated on the six datasets, because the values of *k* and *σ* (a parameter of SC), are different in each dataset. *k* and *σ* have a serious impact on the precision of SCDNN results. Next, the testing datasets are used to compare the performance of the remaining five models.

#### 4.3.1. Efficiency of Varied Cluster Numbers and Values of *σ*

**Figure 3:**
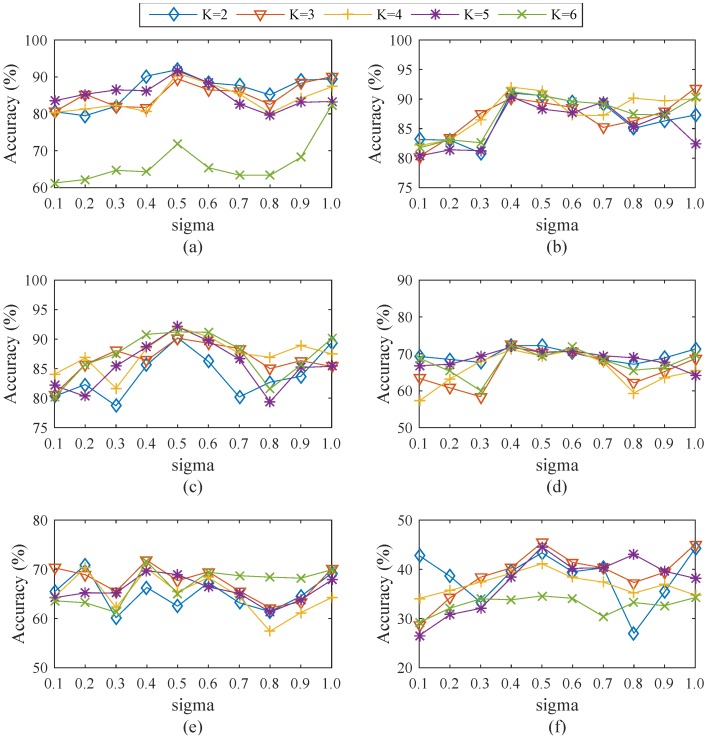
(**a–f**) Comparing SCDNN accuracy over several cluster numbers *k* and *σ* values for the six datasets.

The SCDNN algorithm is composed of three steps. First is clustering the training dataset; the numbers of clusters *k* ranges from 2 to 6, and the value range of *σ* in the SC algorithm is 0.1 to 1.0. The training data subsets are divided based on the values of *k* and *σ*. Different parameters impact the experiment results. In order to obtain the best efficiency, the average detection accuracy rate of the SCDNN is determined for the six datasets. These results are shown in Figure 3.

**Figure 4:**
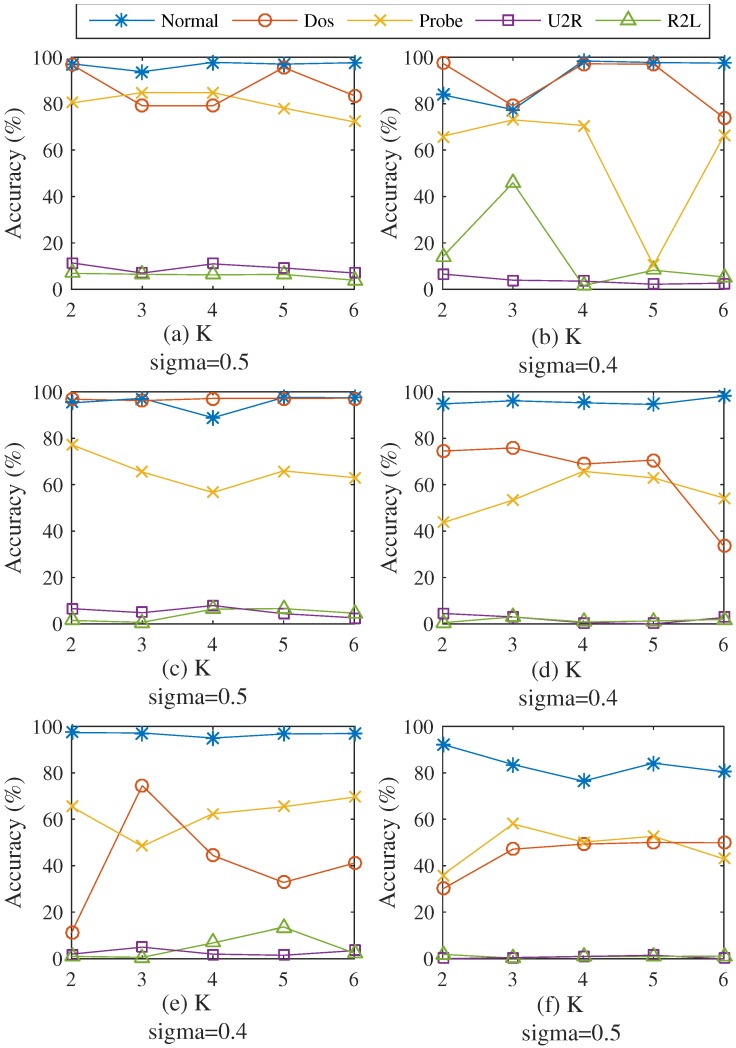
(**a–f**) Comparing SCDNN accuracy for different numbers of clusters *k* for the six datasets.

Figure 3a–f shows testing results from the six datasets obtained from different values of *k* and *σ*. For almost all datasets, SCDNN performs best when *σ* is between 0.4 and 0.5; likewise, the optimal value of *k* is between 2 and 5. In order to clearly explain the testing selection process, the best parameters for the five attack types in each dataset are shown in Figure 4.

SCDNN prediction accuracy of the five network intrusion types with optimum *σ* values are highly volatile with increasing values of *k* in all datasets. A high *k* value can efficiently improve SCDNN accuracy; more clusters can be divided into smaller data subsets, which damages the integrity of the input data features and leads to poor SCDNN performance. U2R and R2L attacks comprise a small proportion of the dataset, which prevents the classifier from learning enough information and causes unbalanced dataset categories. Cluster number can determine how well the particular features of underrepresented attack types are mined; therefore, it is necessary to strikes a balance between the number of cluster and efficiency. The optimal values of *k* for each dataset (that is, those with the best SCDNN accuracy) are presented in Table 2.

Figure 3 and Figure 4 and Table 2 show that accuracy rates are volatile when *k* is less than 4, while great stability can be obtained when *k* is greater than 5. The best results occur when *k* is set to 2 in Dataset 1, with an overall accuracy of 91.97% and high DoS, probe and R2L detection rates. This implies that the clustering process can be classified into normal and attack types, which helps the SCDNN maintain efficient network intrusion categories. In the same way, the best results occur when *k* is 4, 5, 3 and 3 in Datasets 2 to 5, respectively. The total accuracy is 44.55% in the Dataset 6 when *k* is set to 5. Accuracy is low across attack types because attack features are similar to those of normal data and are hard to detect. U2R and R2L attack detection results in low accuracy in all datasets except Dataset 1, probably because there are few such records in the dataset and their features are similar to those of DoS and probe attacks, which provides the DNN with less information and causes increased detection rate errors.

#### 4.3.2. Results and Comparisons

**Figure 5:**
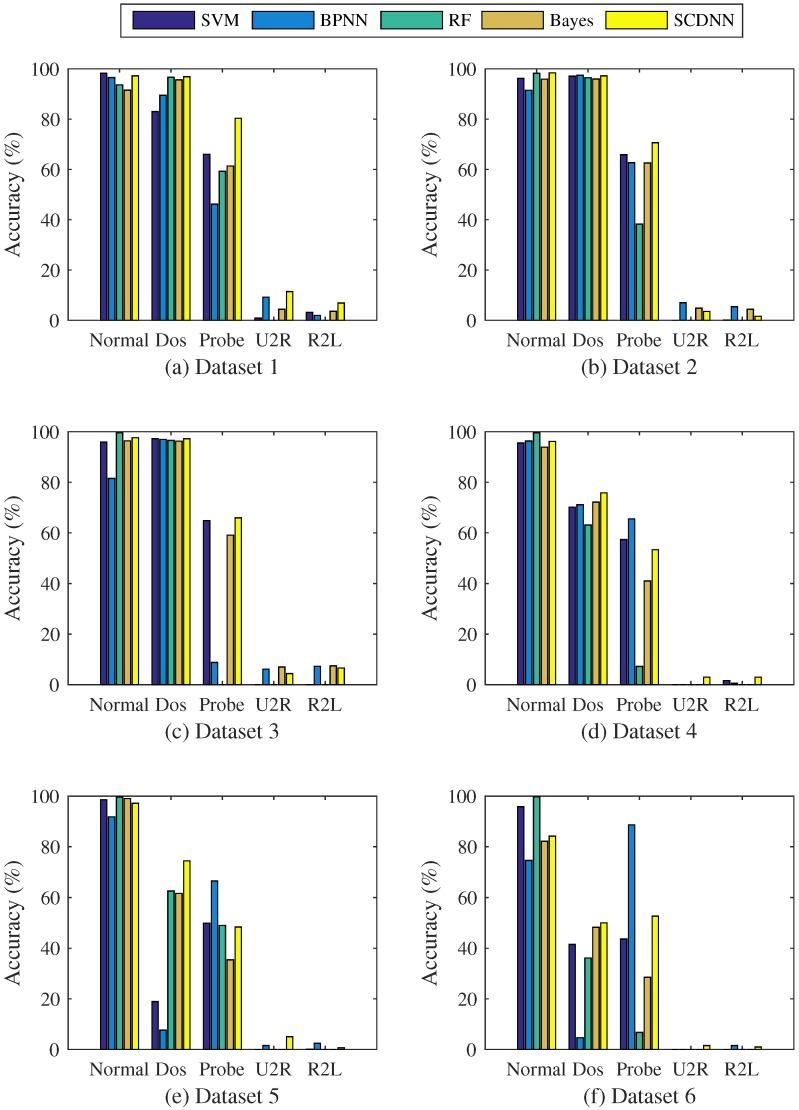
(**a–f**) Prediction accuracy histogram of the five detection models.

In this section, the fusion matrix and evaluation criteria are calculated for the SCDNN and the four traditional detection methods for each of the six datasets. The optimal cluster number *k* and *σ* value are fed into each DNN described in Section 3. The DNNs have 65 input dimensions and five output dimensions, because the original dataset is a 65-dimensional vector and the goal is to distinguish five intrusion attack types. There are two hidden layers, the first has 40 neurons and the second has 20. The softmax layer has five dimensions. 10% validation is used to check for overfitting during the training process. The results of the above experiment are shown in Table 3 and Figure 5.

The records are unbalanced in each of the six datasets; Normal and DoS cases are well-represented, whereas U2R and R2L occur rarely, because the latter two cases require complex intrusion actions generally carried out by an advanced user, resulting in more covert intrusion that is difficult to detect.

From Table 3 and Figure 5, considering overall accuracy, the SCDNN performs better than the other four methods and has the lowest error rates. Moreover, the proposed method shows especially good performance for the sparse U2R and R2L attack types in all datasets, and produces a higher accuracy rate than the other methods. The best accuracy rate for Dataset 1 is 98.21%, obtained by SVM. The SCDNN achieves 97.21% accuracy for normal data, proving that the SVM has better learned the features of the data than other models. All methods are effective for intrusion detection with this dataset, except for the RF method, which has low precision and 0% detection accuracy for U2R and R2L attack types. For Dataset 2, the SCDNN attains higher precision for normal, DoS and probe traffic, with rates of 98.42%, 97.20% and 70.64%, respectively, and lower precision for U2R and R2L traffic than the BPNN. In this experiment the BPNN results in higher recall than other methods for the sparse attack types, which illustrates that the BPNN, with simple layer, has obtained the expected accuracy in a specific dataset, though its performance is unstable. The RF algorithm performs well for the more common traffic, but obtained 0% accuracy in detecting cases of U2R and R2L attacks. It may be that the construction of the trees used by this method can effectively classify most data, but some sparse attack types belong to false classes.

In Dataset 3, the SCDNN performs best on DoS and probe intrusions, with precision rates of 97.23% and 64.96%, respectively. This result indicates that the proposed method is robust and captures most patterns in the different datasets; in addition, the Bayes model has the highest rates, 7.02% and 7.46%, respectively, for the U2R data among all methods. The traditional machine learning models, RF and SVM, produce good results for normal and DoS data. BPNNs, RFs and the SCDNN show good and effective detection rates for normal, DoS and probe data in Datasets 4 and 5; the RF has the highest accuracies, 99.63% and 99.69%, of detecting normal traffic in the two datasets, though the SCDNN produced higher accuracy for DoS data and is effective in detecting sparse attack types. The RF, SVM and Bayes methods performed poorly in classifying rare attack types, with accuracy rates close to 0%.

Overall precision of all methods is quite low for Dataset 6, although the SCDNN performs better than other methods in overall accuracy and recall. Specifically, the RF has the highest accuracy rate, 99.72%, for normal data; the precision of the BPNN for probe traffic is 88.67%, significantly better than the second highest rate, 52.66%, produced by the SCDNN. One potential explanation for this difference is that the BPNN learns enough patterns from the dataset to detect this kind of attack type. In summary, the SCDNN is good at detecting probe intrusions and the sparse attack types, U2R and R2L. It obtains high overall accuracy and recall; however, the SVM, BP, RF and Bayes methods can serve for detecting normal traffic and DoS data. Especially, the RF is excellent in capturing the normal class, producing the highest accuracy rates in Datasets 3–6. These results show that DNNs are robust and capable of detecting sparse attack classes and can locate new attack types, which further proves that SCDNN are more suitable for intrusion detection.

The architecture of the proposed model is a hybrid of spectral clustering technology and deep neural networks. SC clusters samples by their features, an unsupervised process that can classify unknown attack type. Therefore, clustering can find patterns hidden in the samples based their attributes. In Experiment 1, SC is used to divide the dataset; initial groups are obtained using sample attributes. In this way, each sub-DNN is trained with special datasets obtained in the clustering process, and have found more information than they would otherwise, because the DNN model has more layers than the other, shallower models and used SAE to resample the raw dataset. The question of the number of hidden layer to use in the DNN is more complicated, as the DAEs increases computational overhead and finds more robust features in hidden layers. As shown in Table 1, the five attacks types are imbalanced. The last three attack types are the rarest. Their features are obtained by the data subsets generated by the clustering stage. This can help the SCDNN learn more features of sparse attack types. According to Table 3, the SCDNN performs well in detecting sparse attacks types: probe, U2R and R2L.

### 4.4. SCDNN Experiment II with a WSN Dataset

In order to test the performance of SCDNN for maintaining the security of WSNs, in this experiment the all-in-one (ns-2) network simulator platform is used to generate a test dataset. Because WSNs are widely used in industry and their multi-hop, distributed nature differs from that of exit networks, external intrusion in WSNs has become a popular topic of research in academia. Various kinds of security attack cases increase with the number of sensor nodes in a WSN. Therefore, it is important to adopt the proposed method to detect intrusion attacks in WSNs.

In this experiment, there are 10 to 50 sensor nodes to simulate attacker datasets; the ad hoc on-demand distance vector (AODV) protocol is assigned in each nodes. The sensor nodes transport protocol with a constrained bit rate and generated at a random time. The simulated platform generates a dataset which include protocol messages and data messages. For convenience, the dataset is adapted for the proposed algorithm; synthetic data are labelled with security attack types based on known major attack types on the AODV protocol and are shown in Table 4 [50,51].

There are seven attack types that can be categorized based on consideration of their protocol messages in term of message type, transfer time and the number of hops. In this experiment, the detection methods are run 500 times each and the average detection accuracy if given as the final result. The output results are shown in Table 5.

**Figure 6:**
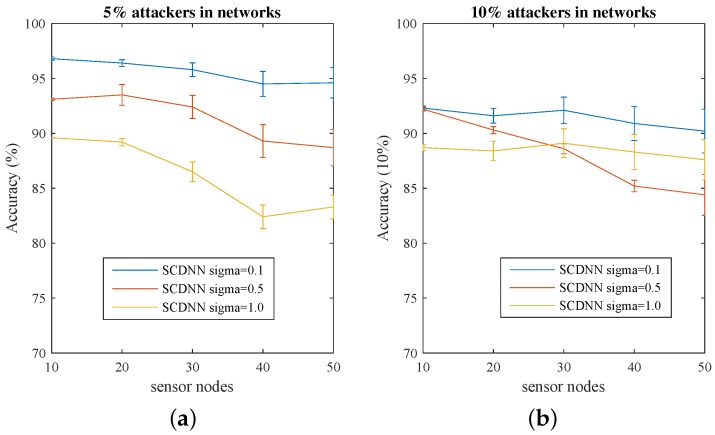
Detection accuracy with five sensor node scales with 95% confidence interval. Accuracy is shown for (**a**) a 5% attacker and (**b**) a 10% attacker, 500 times each.

The detection accuracy at each sensor node scale is shown in Figure 6 and Table 5. The SCDNN can detect all seven attack types with high detection rates. For the 5% attack scale, the proposed model can find almost all attackers. Detection rates decrease with increasing the size of the attacker to 10% in the network. Moreover, detection accuracy is impacted for different values of *σ*. The reason for this change may be that when the attack process attempts to modify router information, the resulting increase of transport packets in WSNs makes detection performance more unstable.

## 5. Discussion

**Figure 7:**
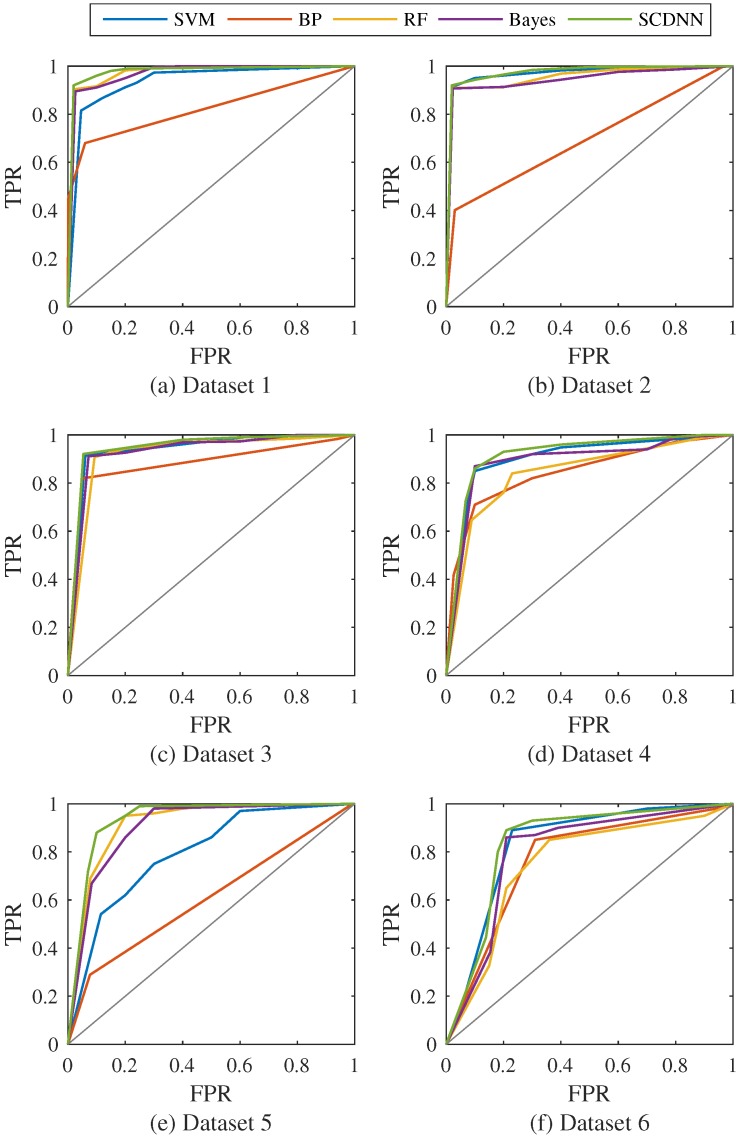
(**a–f**) Receiver operating curves (ROC) curves of the five models in the six datasets, shown with optimal values of *k* and *σ*.

In order to evaluate the performance of the SCDNN and compare it with the experimental results of the other four models in this section, receiver operating curves (ROC) are obtained for all models [52]. The ROC function is widely used to indicate an algorithm’s discriminative capability in this field. The overall category performance of each model, the true positive rate (TPR) from Equation (11) and the false positive rate (FPR) (calculated as 1-Specificity from Equation (13)) are used to generate the ROC in Figure 7.

In this section, there are five network data types to be detected, an example of multiclass classification. Handling five classes is complex, with the possibility of *n* positive results and $n^{2}+n$ errors to consider. In this situation, multiclass ROC and area under curve (AUC) methods are used to plot ROC curves and calculate each AUC [53]. The compared AUC value results for these ROCs in the six dataset are shown in Table 6.

**Figure 8:**
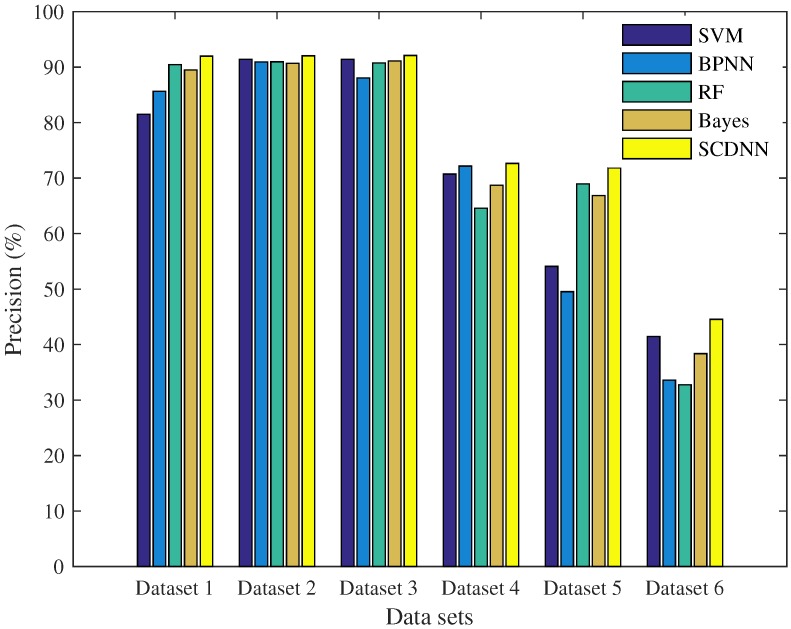
Average precision histograms for the five models compared between the six datasets.

Table 6 and Figure 7 show that the SCDNN has the largest AUC of the five models for the six datasets. This indicates that the SCDNN performed better than other models and can obtain higher detection rates in networks. Overall accuracy is used to generate the histograms and compare the results from the six datasets, as shown in Figure 8. This is a more detailed evaluation of classification performance for all five types (one normal and four attacks).

In real intrusion data, there is an unbalanced distribution among the four attack cases, and the scale of the data is larger [54]. Models in literature are difficult to categorise, so it is important for intrusion detection models to detect sparse attack cases. The results shown in Figure 7 and Figure 8 indicate that the SCDNN produces higher accuracy than other methods in the six datasets; thus, the proposed algorithm performs well on datasets with a range of distributions. In particular, the Bayes algorithm has best recall of 92.57% and the SCDNN method obtains best accuracy of 91.97% in Dataset 1. In Dataset 2, the BPNN has a best recall, of 92.88%, higher than that of the SCDNN method. The reason for this disparity may be that the percentage of attack cases is larger in the training dataset, which leads to the Bayesian and BPNN approaches developing larger probabilities for these active cases. From the above experiment, we see that the SCDNN algorithm is not only good at detecting normal dataflow, as well as DoS and Probe attacks, but also obtained higher accuracy for sparse attack types, U2R and R2L, in the six datasets. The SCDNN model is a suitable approach for intrusion detection in complex networks.

From the above discussion, the problem of selection number clusters is a general problem for all clustering methods [7]. In this paper, the proposed method combined the SC clustering technique and DNN ensemble algorithm; the parameters of the number *k* of clusters and *σ* were obtained from experiment results of six datasets. For other domain datasets, according the distribution of datasets, the range of *k* is set 2 through *n* and the range of *σ* is set 0.4 through 0.6 can be detected the effectiveness of the proposed method, in which *n* is number of categories [28,55]. Otherwise, for a large data set, the time complexity should be a major consideration because it spends more time for processing the training and detecting intrusion network. The novel optimized algorithm is considered for parallel computing and cloud computing.

## 6. Conclusions

In security technology, low-frequency attack events are challenging to predict and can severely threaten networks. As such, this paper puts forward a novel approach that takes advantage of spectral clustering and deep neural networks to detect attack types. In the first stage, network features are captured by clusters and divided into *k* subsets in a bid to discover more knowledge and patterns from similar clusters. In the second stage, highly abstract features are obtained by deep learning networks from the subsets produced during the clustering process.

Finally, testing subsets are used to detect attacks. This is an efficient way to improve detection rate accuracy. Experimental results show that the SCDNN performs better than SVM, BPNN, RF and Bayesian methods, with the best accuracy rates over the six datasets derived from the KDDCUP99 and NSL-KDD. Additionally, the proposed algorithm is more capable of classifying sparse attack cases and effectively improves detection accuracy in real security systems. However, the limitation of the SCDNN is that its weight parameters and the thresholds of each DNN layer need to be optimised, and the *k* and *σ* parameters of the clusters are determined empirically, not through mathematical theory, which deserves further study.

## Figures and Tables

**Table 1:** The distribution of the training and testing sets from the six datasets generated from KDD’99 and NSL-KDD.

Dataset	Training Dataset					Testing Dataset				
	Normal%	DoS%	Probe%	U2R%	R2L%	Normal%	DoS%	Probe%	U2R%	R2L%
Dataset 1	17.96	72.28	7.583	0.096	2.079	19.48	73.90	1.339	0.073	5.205
Dataset 2	19.48	78.40	1.645	0.021	0.451	19.48	73.90	1.339	0.073	5.205
Dataset 3	19.69	79.23	0.831	0.011	0.228	19.48	73.90	1.339	0.073	5.205
Dataset 4	53.38	36.65	9.086	0.044	0.860	43.07	33.08	10.73	0.887	12.21
Dataset 5	48.56	33.11	16.81	0.075	1.435	43.07	33.08	10.73	0.887	12.21
Dataset 6	53.38	36.65	9.086	0.044	0.830	18.16	36.64	20.27	1.688	23.24

**Table 2:** Detection accuracy of five attack types using the optimal number of clusters for each dataset.

Dataset	*k*	*σ*	Nor (%)	DoS (%)	Probe (%)	U2R (%)	R2L (%)	Accuracy (%)
Dataset 1	*k* = 2	0.5	97.21	96.87	80.32	11.4	6.88	91.97
Dataset 2	*k* = 4	0.4	98.42	97.2	70.64	3.51	1.57	92.03
Dataset 3	*k* = 5	0.5	97.61	97.23	65.96	4.39	6.59	92.1
Dataset 4	*k* = 3	0.4	96.17	75.84	53.37	3.00	3.01	72.64
Dataset 5	*k* = 3	0.4	97.19	74.51	48.37	5.00	0.62	71.83
Dataset 6	*k* = 5	0.5	84.20	50.02	52.66	1.50	0.98	44.55

**Table 3:** Comparing network intrusion detection results for the six datasets (%).

Dataset	Model	Normal	DoS	Probe	U2R	R2L	Acc	Recall	ER
Dataset 1	SVM	**98.21**	83	66.01	0.88	3.14	81.52	77.72	18.48
	BP	96.51	89.49	46.18	9.21	1.93	85.66	83.48	14.34
	RF	93.65	96.62	59.27	0	0	90.44	91.08	9.56
	Bayes	91.51	95.59	61.35	4.39	3.56	89.48	**92.57**	10.52
	SCDNN	97.21	**96.87**	**80.32**	**11.4**	**6.88**	**91.97**	91.68	**8.03**
Dataset 2	SVM	96.22	97.1	65.84	0	0.05	91.39	90.52	8.61
	BP	91.44	**97.42**	62.69	**7.02**	**5.41**	90.93	**92.88**	9.07
	RF	98.23	96.48	38.26	0	0	90.95	89.51	9.05
	Bayes	95.92	95.98	62.55	4.82	4.38	90.69	91.07	9.31
	SCDNN	**98.42**	97.2	**70.64**	3.51	1.57	**92.03**	91.35	**7.97**
Dataset 3	SVM	95.87	97.23	64.86	0	0.06	91.41	90.59	8.59
	BP	81.53	96.95	8.81	6.14	7.26	88.03	90.05	11.97
	RF	**99.57**	96.57	0	0	0	90.76	89.37	9.24
	Bayes	96.38	96.29	59.15	**7.02**	**7.46**	91.12	90.95	8.88
	SCDNN	97.61	**97.23**	**65.96**	4.39	6.59	**92.1**	**92.23**	**7.9**
Dataset 4	SVM	95.54	70.18	57.37	0	1.63	70.73	53.26	29.27
	BP	96.35	71.17	**65.55**	0	0.58	72.16	**57.79**	27.84
	RF	**99.63**	63.11	7.23	0	0	64.57	40.45	35.43
	Bayes	93.9	72.18	41.02	0	0	68.73	52.78	31.27
	SCDNN	96.17	**75.84**	53.37	**3**	**3.01**	**72.64**	57.48	**27.36**
Dataset 5	SVM	98.57	18.93	49.89	0	0.11	54.1	20.45	45.9
	BP	91.79	7.63	**66.58**	1.5	**2.43**	49.53	27.56	50.47
	RF	**99.69**	62.64	48.99	0	0	68.93	46.43	31.07
	Bayes	99.06	61.65	35.4	0	0	66.87	44.28	33.13
	SCDNN	97.19	**74.51**	48.37	**5**	0.62	**71.83**	**55.08**	**28.17**
Dataset 6	SVM	95.81	41.5	43.67	0	0	41.46	30.6	58.54
	BP	74.72	4.61	**88.67**	0	1.53	33.59	30.6	66.41
	RF	**99.72**	36.15	6.74	0	0	32.73	18.9	67.27
	Bayes	82.16	48.25	28.52	0	0	38.37	30.08	61.63
	SCDNN	84.2	**50.02**	52.66	**1.5**	**0.98**	**44.55**	**37.85**	**55.45**

**Table 4:** Basic attacker types with packet routing protocols on the ad hoc on-demand distance vector (AODV) protocol in wireless sensor networks (WSNs).

Attack Name	Attack Description	Attack Type
Active Reply	The route reply is forged with abnormal support to reply.	1
Route drop	The routing packets are dropped with some specific address.	2
Modify Sequence	The number of target sequences increases with largest maximal values.	3
Rushing	Rushing of routing messages.	4
Data Interruption	A data packet is used to drop the route.	5
Route Modification	The route is modified in Routing Table Entries.	6
Change Hop	The route cost in routing tables entries is altered.	7

**Table 5:** Average detection accuracy for five sensor nodes scales by the SCDNN algorithm using optimal *k* and sigma values (%).

Dataset	Parameter	Sensor Nodes				
		10 Nodes	20 Nodes	30 Nodes	40 Nodes	50 Nodes
5% Attacker in Networks	k=4,σ=0.1	96.8	96.4	95.8	94.5	94.6
	k=4,σ=0.5	93.1	93.5	92.4	89.3	88.7
	k=4,σ=1.0	89.6	89.2	86.5	82.4	83.3
10% Attacker in Networks	k=4,σ=0.1	96.8	96.4	95.8	94.5	94.6
	k=4,σ=0.5	93.1	93.5	92.4	89.3	88.7
	k=4,σ=1.0	89.6	89.2	86.5	82.4	83.3

**Table 6:** Area under curve (AUC) values for the ROCs of each model in the six datasets.

Dataset	SVM	BP	RF	Bayes	SCDNN
Dataset 1	0.88	0.82	0.94	0.93	0.95
Dataset 2	0.95	0.78	0.94	0.94	0.95
Dataset 3	0.95	0.88	0.94	0.94	0.95
Dataset 4	0.82	0.72	0.78	0.80	0.83
Dataset 5	0.71	0.61	0.80	0.79	0.82
Dataset 6	0.61	0.56	0.58	0.61	0.65

## Abbreviations

The following abbreviations are used in this manuscript:

SVM: Support Vector Machine
SOM: Self-Organizing Map
ANN: Artificial Neural Networks
RF: Random Forest
DNN: Deep Neural Network
PSO: Particle Swarm Optimization
KNN: *K*-Nearest Neighbour
IDS: Intrusion Detection System
SGD: Stochastic Gradient Descent
DoS: Denial of service
R2L: Remote to Local
U2R: User to Root
ER: Error Rate
TN: True Negative
TP: True Positive
FP: False Positive
FN: False Negative
TPR: True Positive Rate
FPR: False Positive Rate
BPNN: Backpropagation Neural Network
SCDNN: Spectral Clustering and Deep Neural Network
SC: Spectral Clustering
SAE: Sparse Auto-Encoder
DAEs: Denoising Auto-Encoders
KL: Kullback-Leibler
NJW: Ng-Jordan-Weiss
WSN: Wireless Sensor Network
ROC: Receiver Operating Curves
AUC: Area under the ROC Curve
DBN: Deep Belief Networks
GA: Genetic Algorithms
STL: Self-Taught Learning
DRBM: Discriminative Restricted Boltzmann Machine
AODV: Ad hoc On-demand Distance Vector
